# *In utero* infection of Zika virus leads to abnormal central nervous system development in mice

**DOI:** 10.1038/s41598-019-43303-6

**Published:** 2019-05-13

**Authors:** Wei Zhang, Yong Wah Tan, Wan Keat Yam, Haitao Tu, Lifeng Qiu, Eng King Tan, Justin Jang Hann Chu, Li Zeng

**Affiliations:** 1 Neural Stem Cell Research Lab, Research Department, National Neuroscience Institute, Singapore, 308433 Singapore; 2 Collaborative Translation Unit for HFMD, Institute of Molecular and Cell Biology, Agency of Science, Technology & Research (A STAR), Singapore, 138673 Singapore; 3 Research Department, National Neuroscience Institute, SGH Campus, Singapore, 169856 Singapore; 4 Department of Neurology, National Neuroscience Institute, SGH Campus, Singapore, 169856 Singapore; 5 Neuroscience & Behavioral Disorders Program, DUKE-NUS Graduate Medical School, Singapore, 169857 Singapore; 6 Laboratory of Molecular RNA Virology and Antiviral Strategies, Department of Microbiology and Immunology, Yong Loo Lin School of Medicine, National University of Singapore, Singapore, 117597 Singapore; 7 Lee Kong Chian School of Medicine, Novena Campus, 11 Mandalay Road, Singapore, 308232 Singapore

**Keywords:** Viral infection, Epidemiology, Central nervous system infections

## Abstract

The World Health Organization has declared ZIKA virus (ZIKV) a global public health emergency, prompted by the association of ZIKV infections with severe brain abnormalities in the human fetus. ZIKV preferentially targets human neuronal precursor cells (NPCs) in both monolayer and cortical brain organoid culture systems and stunts their growth. Although ZIKV is well recognized to cause microcephaly, there is no systematic analysis to demonstrate the effect of ZIKV on central nervous system (CNS) development, including brain malformations and spinal cord dysfunction. Here, we conducted a longitudinal analysis to show that a novel mouse model (infected *in utero* and monitored after birth until adulthood) recapitulates the effects of ZIKV infection affecting neural stem cells fate and leads to a thinner cortex and a smaller brain. Furthermore, we demonstrate the effect of ZIKV on spinal cord function. Specifically, we found significant reductions in neuron numbers in the anterior horn of grey matter of the spinal cord and muscle dystrophy with a significant decrease in forepaw grip strength in the ZIKV group. Thus, the established mouse model of ZIKV infection leading to abnormal CNS development will help to further advance our understanding of the disease pathogenesis.

## Introduction

ZIKA, its name came from the ZIKA Forest of Uganda, where the virus was first isolated in 1947 from a rhesus macaque monkey^1^. It was not until 1954 that the isolation of ZIKA from a human was published^2^. ZIKA virus (ZIKV) is a member of the virus family Flaviviridae and the genus Flavivirus. It is spread by daytime-active Aedes mosquitoes. ZIKV can also spread from a pregnant woman to her fetus^3^. This can result in microcephaly, a severe brain malformation, and other birth defects^4^. ZIKA infections in adults may also result in Guillain–Barré syndrome (GBS)^5^, a neurological disorder. Ever since the outbreak of ZIKA in Brazil in early February 2016, as of July, the virus has spread to 63 countries, of which 13 are reporting microcephaly cases and 15 cases of GBS (World report).

The suspected link between infection by ZIKV and microcephaly is an urgent global health concern. Studies suggested that the SOX2^+^ neural progenitor cells (NPCs) are the directed target of ZIKV infection in human brain organoids^6,7^. ZIKV stock produced from the infected *rhesus Macaca* cell line LLC-MK2 can efficiently infects human neural progenitor cells (hNPCs) derived from induced pluripotent stem cells. Infected hNPCs further release infectious ZIKV particles. Importantly, ZIKV infection increases cell death and dysregulates cell-cycle progression, resulting in attenuated hNPC growth^8^. Not only the human progenitors *in vitro*, recent studies also showed that cerebral organoids can recapitulate key features of human cortical development, including neurogenesis and notably, a distinct human specific outer radial glial cell layer-derived from human iPSCs. ZIKV infection leads to increased cell death and reduced proliferation, resulting in decreased neuronal cell-layer volume resembling microcephaly^6^. In addition, Garcez *et al*.^7^ investigated the effects of ZIKV infection in human neural stem cells growing as neurospheres and brain organoids. They found that ZIKV reduces cell viability and growth in neurospheres and brain organoids. All these findings suggest that ZIKV abrogates neurogenesis during human brain development. However, how ZIKA enters the brain remains unknown. Once ZIKV crosses the brain blood barrier (BBB), it may enter through specific transmembrane receptors AXL into NPCs^9,10^. Using single-cell RNA-seq and immunohistochemistry, Nowakowski *et al*.^10^ found that viral entry receptor AXL is highly expressed in human radial glial cells, astrocytes, endothelial cells, and microglia in developing human cortex. Furthermore, AXL expression is conserved in rodents and human cerebral organoid model systems^10^, suggesting neural stem cells are more susceptible to ZIKV infection.

Precise timing of proliferation/self-renewal of NPCs and their differentiation, neuronal migration and maturation are essential for normal mammalian brain development. Disruptions of this process will lead to brain development disorder, including microcephaly. Li *et al*.^11^ established a ZIKV infection model in mice via *in utero* injection during embryonic development and found ZIKV infection causes microcephaly. Specifically, they found that ZIKV infection leads to cell-cycle arrest, apoptosis, and inhibits NPCs differentiation, resulting in thinner cortex during embryonic cortical development. However, they failed to develop the mouse model that infects ZIKV at the embryonic stage which can allow the mice to survive postnatal till adult stage.

Later on, another group showed that ZIKV can affect adult brain and causes major, lasting damage^12^. Experiments on adult mice with triple knockout IFN regulatory factor (IRF) showed that ZIKV seems to attack neural stem cells (NSCs) at the subventricular zone (SVZ) of the forebrain and the sub-granular zone (SGZ) of the hippocampal dentate gyrus (DG)^12^. ZIKV infection affects adult neurogenesis in the hippocampal DG, leads to cell death, and reduces proliferation, resulting in an Alzheimer’s-like effect in adult mice^12^. The NSCs in the hippocampal dentate gyrus, the profound neurogenic niches area^6,13,14^, are vital to learning and memory, and losing of these NSCs could have disastrous effects. Impaired adult neural stem cells function is linked to depression-like behaviour in schizophrenia^15,16^ and impairs neurogenesis, implicating cognitive decline in Alzheimer’s disease^17,18^. However, most microcephaly cases indicate that the infection by Zika virus is at the pregnancy stage.

Although the effect of ZIKV on embryonic brain during pregnancy and adult brain development has been studied separately in mice^11,12^, its effect on central nervous system (CNS) throughout the whole lifespan from pregnancy to adulthood has not been studied. In this study, we conducted a systematic and longitudinal analysis to extend our understanding of the potential of ZIKV infection on CNS development, including brain malformations and spinal cord dysfunction. We have developed a novel mouse model (infected *in utero* and monitored after birth until adulthood) that recapitulates the effects of ZIKV infection, which affects NSCs proliferation, differentiation, and death during cortical development, and eventually leads to a thinner cortex and a smaller brain. Our model helps to explain how ZIKV causes brain malformations and microcephaly. Additionally, we demonstrate the effect of ZIKV on spinal cord development which lead to postnatal muscle defects and paralysis, adding our knowledge on how ZIKV exposures *in utero* leads to CNS defects. The established mouse model of ZIKV infection leading to abnormal CNS development will help further improve our understanding of the disease pathogenesis and provide an accessible platform for modelling CNS development for testing treatments, including potential ZIKV antiviral drugs and vaccines against congenital ZIKA syndrome (CZS).

## Results

### ZIKA infection causes abnormal brain development

**Figure 1:**
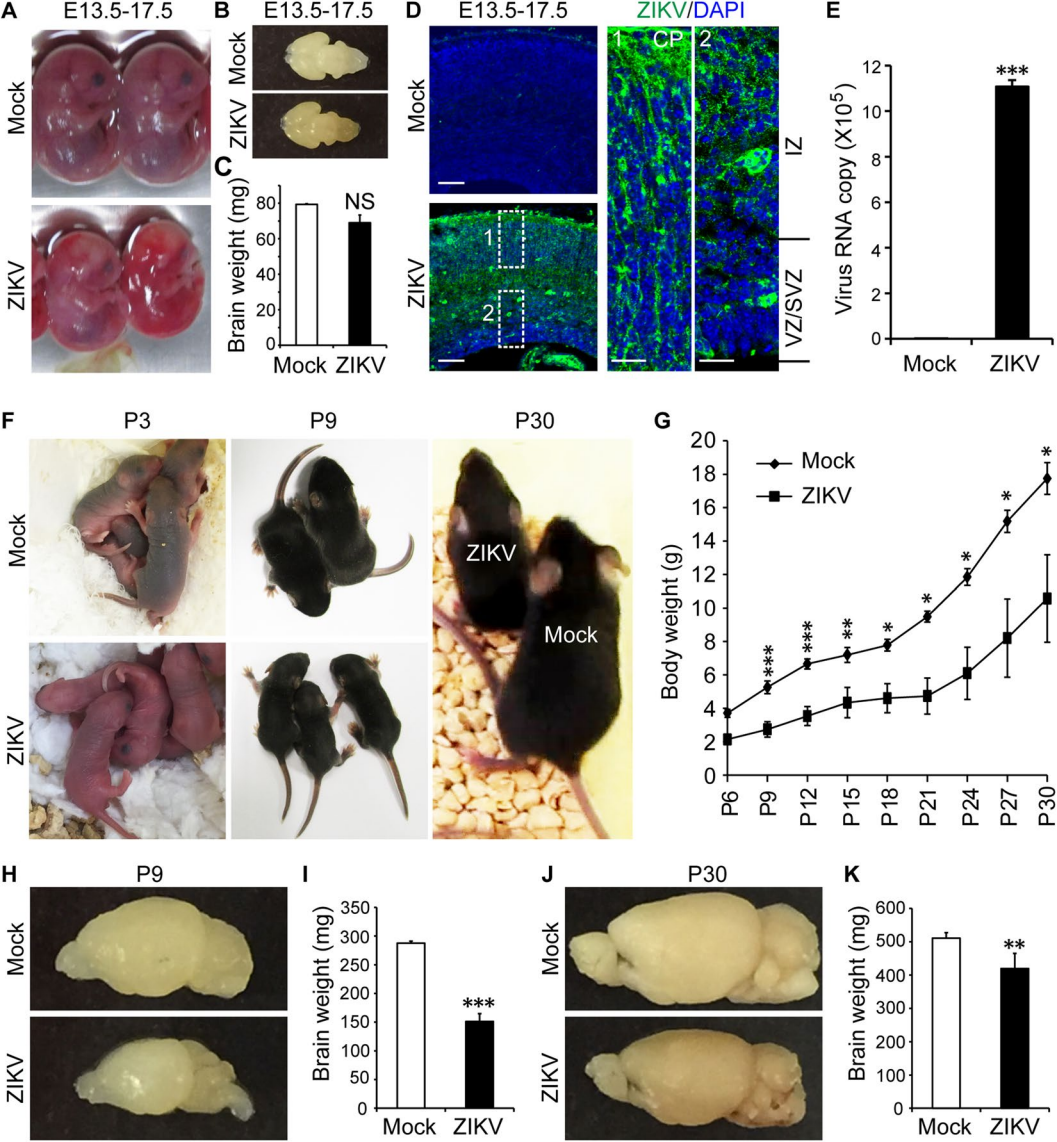
ZIKV infection during pregnancy induces mouse brain development disorder after birth. ZIKV (500 PFU) and mock infection were conducted via *in utero* at E13.5. (**A**) E17.5 embryos morphology post- ZIKV and mock infection. (**B**) Brain images at E17.5. (**C**) brain weights at E17.5 (n = 3 per group). (**D**,**E**) ZIKV detection in the embryo cortex by immunostaining (**D**) and qPCR (**E**, n = 3–4 per group). Scale bar: left panels, 50 µm; enlarged images in the right panels, 10 µm. (**F**) Pups at P3, P9 and P30 and (**G**) changes in their body weights post-ZIKV infection (n = 5, ZIKV 500 PFU) at different postnatal time points. (**H**) Brain image and (**I**) brain weight at P9 after ZIKV infection (500 PFU, n = 5–6 per group). (**J**) Brain images and (**K**) brain weight at P30 after ZIKV infection (500 PFU, n = 6 per group). *P < 0.05, **P < 0.01, ***P < 0.001, calculated by Student’s *t*-test. NS, not significant. Graphs indicate mean ± SD.

Zika virus (ZIKV) infection increases cell death and dysregulates cell-cycle progression, resulting in attenuated human neural progenitor cells growth^8^. To determine the effect of ZIKV on brain development in neonatal mice, the uterine horns of E13.5 mice were exposed by laparotomy. 500 PFU of ZIKV (Strain: PRVABC59, Source: Puerto Rico) or culture medium (mock) were then injected through the uterine wall into one of the lateral ventricles (LV) of each embryo with a glass capillary. The uterine horns were placed back into the abdominal and the embryos were allowed to continue with normal growth. Four days post-ZIKV injection at E17.5, we found that ZIKV-infected embryos showed smaller brain morphology compared to the mock group (Fig. 1A), but the brain weight of the ZIKV group showed no significant changes (Fig. 1B,C). The infection of the embryonic brains was verified by immunohistochemistry (IHC) staining with an antibody against ZIKV (Fig. 1D). Immunostaining showed that ZIKV was able to infect the brain cells located in the VZ, SVZ, and cortical plate (CP) (Fig. 1D). Real-time PCR confirmed the significant increase in virus RNA copy in these ZIKV-infected brains (Fig. 1E).

At the postnatal stage, we first examined the body weights of neonates post-ZIKV infection. We found that ZIKV-infected pups exhibited smaller body sizes and body weights than mock pups from postnatal day 3 (P3) to P30 (Fig. 1F,G). Furthermore, we found that infection with 2000 to 20,000 PFU of ZIKV caused pups death in a dose-dependent manner within two weeks post-ZIKV infection, with most pup’s death at the highest doses (Supplementary Fig. 1A). In addition to mock infection, dengue virus (DENV) was also used in our experiments to compare with ZIKV infection. We observed that the pup’s survival ratio was lower with ZIKV than DENV infection at 500 PFU (Supplementary Fig. 1A). The body weights of the surviving ZIKV-infected pups were also lower than those of the age-matched pups injected with DENV or mock solution; and no significant difference in body weights exist between the DENV and mock group (Supplementary Fig. 1B). Next, we examined the brain weights of the ZIKV- and DENV-infected groups. We found that at P9 (Fig. 1H,I) and P30 (Fig. 1J,K), the brain weights of the ZIKV group were significantly lower (P9, ~50% reduction; P30, ~18% reduction) than those of the mock group. However, the weights of the brains infected with DENV were not significantly different from those in the mock group at P9 (Supplementary Fig. 1C,D). We also investigated the ZIKV infection efficiency among different tissues at P9. Q-PCR demonstrated that the highest expression of ZIKV was in the brain and spinal cord, with less expression in the lung, liver, and small intestine (Supplementary Fig. 1E). Taken together, our results demonstrated that ZIKV but not DENV infection can affect brain development in the VZ, SVZ, and CP region and reduces body and brain weight at the embryonic stage in mice. This also suggests that ZIKV infection induces abnormal brain development at embryonic stage.

### ZIKV impairs NSCs neurogenesis during neocortex development

**Figure 2:**
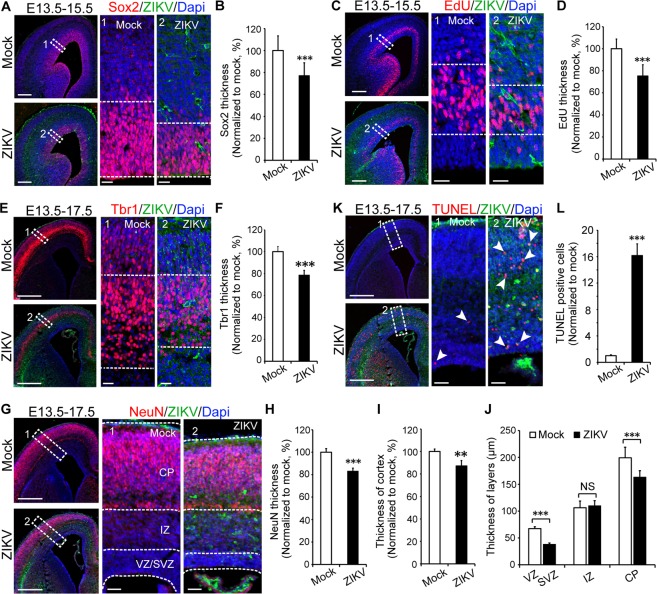
ZIKV impairs NSCs proliferation, differentiation, and leads to cell death and small brain cortices during neocortex development. ZIKV (500 PFU) and mock infection were conducted via *in utero* at E13.5. (**A**) Sox2 staining and thickness calculation (**B**, n = 3 per group) of brain sections at E15.5. (**C**) EdU-labelled NSCs and thickness calculation (**D**, n = 3 per group) at E15.5. (**E**) Immature neuron marker Tbr1 staining and thickness calculation (**F**, n = 5–6 per group) at E17.5. (**G**) NeuN staining of brain section at E17.5 and thickness calculation of NeuN staining (**H**), the entire cortex (**I**), and different layers of the cortex (**J**, n = 5–6 per group). (**K**) TUNEL staining and quantification (**L**, n = 5–6 per group, >500 cells were counted) in brain sections at E17.5. **P < 0.01, ***P < 0.001, calculated by Student’s *t*-test. NS, not significant. Graphs indicate mean ± SD. Scale bar: A, C, E, G, and K, left panel, 200 µm; enlarged images in the right panel, 20 µm.

Given that ZIKV infection induces abnormal brain development, next, we analysed the effect of ZIKV infection on neural stem cells during cortical development. To determine the effect of ZIKV on NSCs proliferation, we injected ZIKV into the lateral ventricle zone at E13.5. Two days later at E15.5, brain tissues were harvested and immunostained with Sox2, a neural stem cell marker. We measured the thickness of the Sox2-positive area in the subventricular zone-ventricular zone (SVZ-VZ) and found the Sox2-positive area to be 23% thinner in the ZIKV-infected brain than mock group (Fig. 2A,B). By immunostaining of the NSCs division marker histone H3, we found that compared to the mock group, the ZIKV-infected group exhibited a 20% decrease in phosphor-histone H3-positive cells in the SVZ (Supplementary Fig. 2A,B). To determine the effect of ZIKV on NSCs proliferation, we administered an intraperitoneal injection of EdU (5-ethynyl-2′-deoxyuridine) to pregnant mice two hours before brain tissue collection and immunostained for EdU. EdU is a thymidine analogue which is incorporated into the DNA of dividing cells. It is widely used to track newly generated progenitor cells undergoing proliferation^19^. We found the EdU-positive area to be 25% thinner in the ZIKV-infected brain than in control (Fig. 2C,D). In addition, immunostaining of another proliferation marker Ki67 demonstrated that NSCs proliferation was decreased to 72% relative to control after ZIKV infection (Supplementary Fig. 2C,D). Our results indicate impaired NSCs proliferation in ZIKV infection in mice.

Next, we examined whether ZIKV infection affects neural differentiation. Four days post-ZIKV infection, we immunostained brain slices at E17.5 with antibodies against TBR1, an immature neuron marker^20^, and NEUN, a mature neuron marker^21,22^. TBR1 and NEUN staining of the cortex of the CP zone showed that ZIKV led to up to a 22% and 17% decrease in TBR1 (Fig. 2E,F) and NEUN-positive areas (Fig. 2G,H), respectively. Our results indicate that ZIKV infection results in thinner cortical layers and fewer neuronal cells. Indeed, immunohistochemistry staining of NEUN followed by measurements of the cortical thickness at different layers revealed that the entire cortex thickness was decreased to approximately 87% at E17.5 post-ZIKV infection compared to mock group (Fig. 2G,I). Specifically, we found that the VZ-SVZ zone and CP zone were both significantly thinner (~45% reduction and ~20% reduction respectively) in the ZIKV group than in the mock group (Fig. 2G,J).

Increased cell death is also one of the causes of microcephaly^12,23^. We conducted a TUNEL assay on the brain slices to determine whether cell death contributes to the smaller size of the infected brains. Indeed, we found enhanced immunostaining signals in the ZIKV-infected cortices compared to that in the mock (Fig. 2K,L). A similar result was obtained when brains were immunostained for the apoptosis marker cleaved caspase-3 (Supplementary Fig. 2E,F), suggesting that ZIKV infection leads to cell death.

To further confirm the effect of small cortices on the development is specific to ZIKV, we conducted a similar analysis on mice brains infected with DENV. Compared to the mock group, the DENV-infected group exhibited no obvious change in NEUN-positive area (Supplementary Fig. 2G,H), whole cortex thickness (Supplementary Fig. 2G,I), or cortical layer thickness (Supplementary Fig. 2G,J). By TUNEL assay, we found no cell death in DENV infected brain, unlike ZIKV infected brain with obvious cell death signals (Supplementary Fig. 2K).

Taken together, our results demonstrate that *in utero* ZIKV infection decreases NSCs proliferation and differentiation, induces cell death and finally, reduces the thickness of the brain cortex, resulting in the formation of microcephaly; whereas, DENV has no such effects.

### ZIKV impairs postnatal brain and young adult neurogenesis

Few reports have been given on the effects of ZIKV *in utero* infection on postnatal brain development in mice. To explore the effect of ZIKV infection on mice postnatal brain development, we injected mock solution and ZIKV into the embryo brain at E13.5. After birth, we monitored the survival and brain development of the pups and further conducted neurogenesis and behavioural studies.

Our previous results showed that at P9, the brain weight was significantly reduced and brain size is smaller in the ZIKV group than in the mock group (Fig. 1F–I). Here, further detailed analysis revealed that the changes of brain weight and size were associated with reduced thickness and neuron density of the CP zone (Supplementary Fig. 3A–C). Specifically, compared to the mock group, the ZIKV-infected group exhibited up to a 50% reduction in NEUN-positive cell density at P9 (Supplementary Fig. 3A–C). Moreover, IHC staining with NEUN revealed that the structural development of the hippocampus was also damaged after ZIKV infection (Supplementary Fig. 3D). The structure changes of hippocampus were also confirmed by Nissl staining (Supplementary Fig. 3E). Additionally, cell death was detected by TUNEL staining in the P9 brain slices of the ZIKV group (Supplementary Fig. 3F).

**Figure 3:**
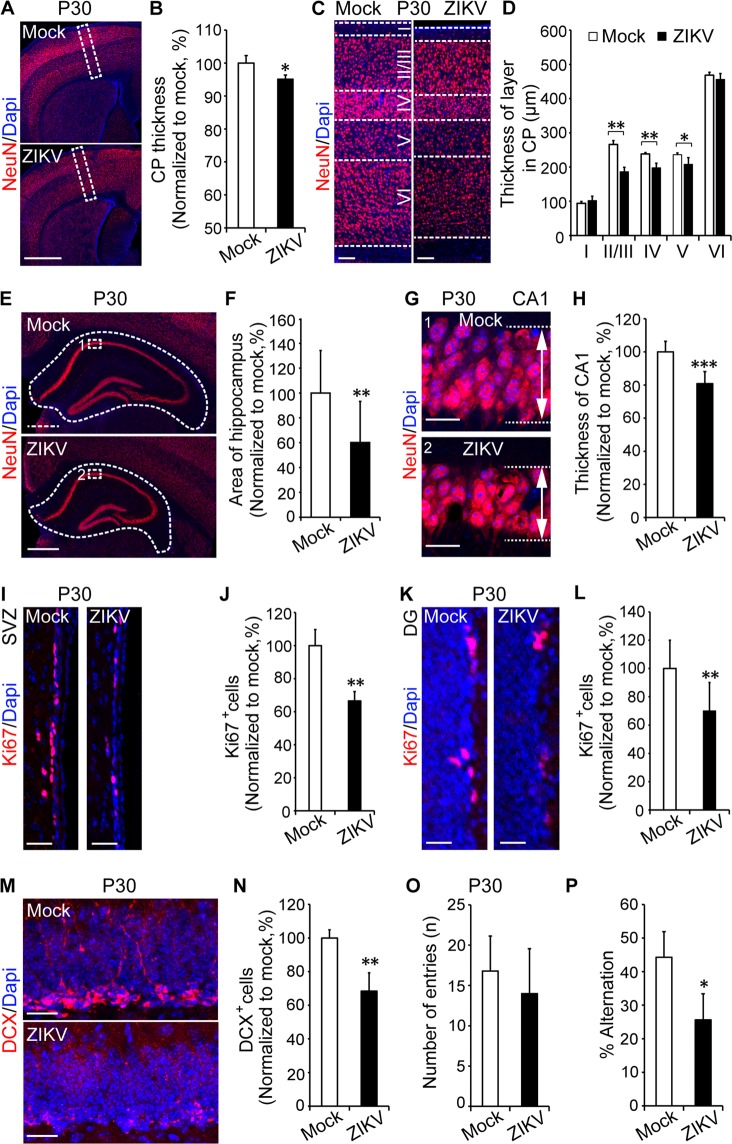
ZIKV leads to postnatal brain reduction and impairs young adult neurogenesis. ZIKV (500 PFU) and mock infection were conducted via *in utero* at E13.5. (**A**) NeuN staining and thickness calculation (**B**, n = 3–5 per group) of brain sections at P30. (**C**) Images show NeuN staining of five layers of the cortex and thickness analyses (**D**, n = 3–5 per group). (**E**) Images show the hippocampus, the area calculation (**F**) and the CA1 image (**G**) and thickness (**H**, n = 3–5 per group) at P30. (**I**) Ki67 staining (**J**) and quantification of positive cells in the SVZ (**J**) and Ki67 staining (**K**) and quantification of positive cells in the DG (**L**, n = 3–5 per group). >1000 cells were counted in SVZ; >150 cells were counted in DG at P30. (**M**) DCX staining and quantification of DCX-positive cells (**N**, n = 3–5 per group, >500 cells were counted) at P30 in hippocampus DG. (**0**,**P**) Spontaneous Y-maze test at P30 (n = 3–5 per group). *P < 0.05, **P < 0.01, calculated by Student’s *t*-test. NS, not significant. Graphs indicate mean ± SD. Scale bar: A, 1 mm; C, 100 µm; E, 500 µm; G, I, K, and M, 20 µm.

At P30, both NEUN immunostaining (Fig. 3A,B) and Nissl staining (Supplementary Fig. 3G–I) revealed that the thickness of the CP was decreased in the ZIKV group compared to that in the mock group. We further define the thickness of the different CP zone layers and found that layers II to V were smaller in the ZIKV group than in the mock group (Fig. 3C,D). In addition to the reduction in the CP zone of the cortex, NEUN immunostaining revealed that the hippocampal area in the ZIKV-infected group was up to 40% smaller than that in the mock group (Fig. 3E,F). Specifically, the thickness of the CA1 was up to 20% smaller after ZIKV infection than without infection (Fig. 3G,H).

Our study demonstrated that the reduction in brain size persisted throughout the postnatal stage post-ZIKV infection and was associated with fewer neuronal cells (NEUN-positive cells) in both the cortex and hippocampal areas.

Given that neurogenes is also occurs in the adult brain, in which there are two neurogenic niche areas at SVZ and hippocampal dentate gyrus^13,14^, we next examined the effect of ZIKV infection on neurogenesis. Sox2 (neural stem cells marker) and Ki67 (proliferation marker) immunostaining showed that in both the SVZ and hippocampal DG areas, the NSCs pool was decreased post-ZIKV infection (Supplementary Fig. 3J,K, 40% decrease; Supplementary Fig. 3L,M, 35% decrease respectively). Similar to the NSCs pool, NSCs proliferation indicated by Ki67 staining showed a 35% reduction in both the SVZ (Fig. 3I,J) and DG (Fig. 3K,L) of the hippocampus in the ZIKV group. We next performed doublecortin (DCX) staining to detect immature neurons in the DG. DCX is a microtubule associated protein expressed by neural progenitor cells and immature neurons. The expression level of DCX in adult brain reflects neurogenesis^24^. Compared to the mock group, the ZIKV group expressed 70% DCX-positive cells in the DG of the hippocampus (Fig. 3M,N). Hippocampus-dependent neurogenesis is associated with learning and memory^25,26^. To test the effect of ZIKV infection on learning and memory, the spontaneous Y-maze test was conducted. Result showed a decreased alternation after ZIKV infection compared to the mock group (Fig. 3O,P), suggesting a cognitive deficit in ZIKV-infected mice.

Therefore, our results demonstrated that ZIKV infection during pregnancy induces a deduction in young adult neurogenesis in the SVZ and DG regions, and leads to cognition impairment.

### ZIKV leads to spinal cord development dysfunction

Our previous results showed that in addition to the brain, high ZIKV RNA copy is also found in the spinal cord of the P9 mouse (Supplementary Fig. 1E). Here, by monitoring the postnatal status of the pups, we found that some ZIKV-infected pups were weaker and smaller in appearance than those in the mock group and exhibited some degree of paralysis (Supplementary Fig. 4A). To determine whether ZIKV infection leads to abnormal spinal cord development, we conducted fluorescent Nissl staining of spinal cord sections. We found that compared to the mock group, the ZIKV-infected group showed approximately 50% Nissl-positive neurons in the anterior horn of the grey matter (Supplementary Fig. 4B,C). We also found cell death in the spinal cord after ZIKV infection by TUNEL staining (Supplementary Fig. 4D,E). Furthermore, HE staining revealed that the hind limb muscle became thinner, and the gap between the muscles increased with ZIKV infection (Supplementary Fig. 4F).

**Figure 4:**
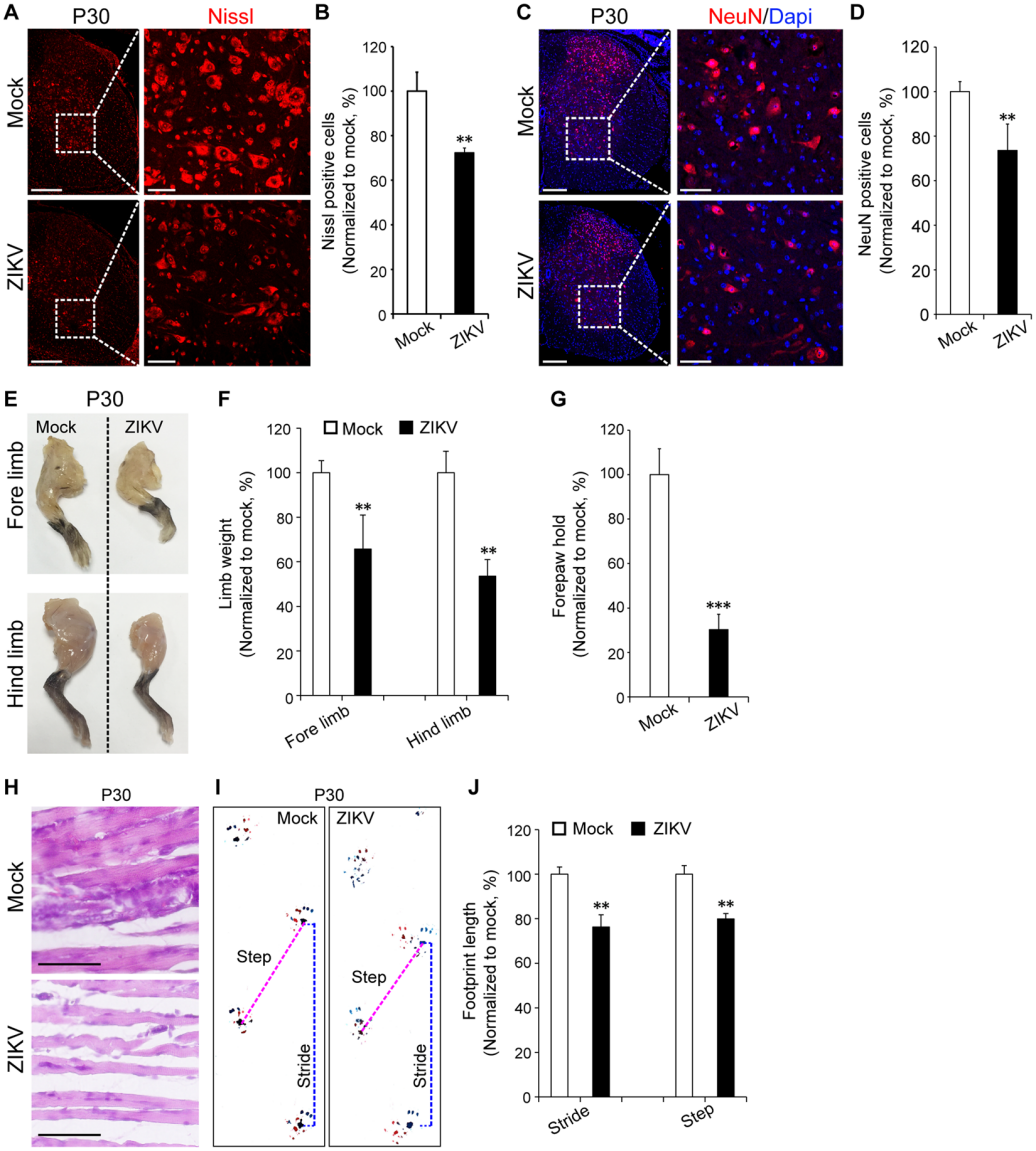
ZIKV infection during pregnancy via *in utero* impairs spinal cord development and function. ZIKV (500 PFU) and mock infection were conducted via *in utero* at E13.5. (**A**) fluorescent Nissl staining and quantification of positive cells in the anterior horn of the grey matter (**B**, n = 3–5 per group, >500 cells were counted) at P30. (**C**) NeuN staining and quantification of positive cells (**D**, n = 3–5 per group, >500 cells were counted) at P30. (**E**) Images of mouse limbs, weights (**F**), and forepaw grip analyses (**G**, n = 3–5 per group) at P30. (**H**) HE staining of hind limb muscle at P30. (**I**) Images of footprints and analysis at P30 (**J**, n = 3–5 per group). **P < 0.01, ***P < 0.001, calculated by Student’s *t*-test. Graphs indicate mean ± SD, Scale bar: A, C, left panel, 200 µm; enlarged images, 50 µm. H, 20 µm.

At P30, we found significantly fewer neurons in the anterior horn grey matter of ZIKV-infected mice than mock-treated mice through both fluorescent Nissl (Fig. 4A,B, 28% decreased) and NEUN staining (Fig. 4C,D, 25% decreased). The weights of the fore limb and hind limb at P30 were decreased in the ZIKV group, and the fore paw grip strength of the ZIKV group was only approximately 70% of that of the mock group (Fig. 4E–G). Similar to the hind limb muscle at P9, the hind limb muscle also showed a change in ZIKV infection (Fig. 4H). Foot print testing revealed that both the stride and step were decreased in the ZIKV group compared to those in the mock group at P30 (Fig. 4I,J). We also compared the pathological changes in other organs such as the lung, liver, heart, and kidney by HE staining, but there was no significant difference between the ZIKV and mock group (Supplementary Fig. 5). Together, our data demonstrated a structural and functional damage to the spinal cord in ZIKV-infected mice, and this is associated with the movement disorder.

## Discussion

Congenital Zika syndrome is a serious birth defect caused by Zika virus infection during pregnancy in humans^3–5^. Some of those with CZS show severe microcephaly and specific brain damage during cortical development^23,27^. Recent studies have shown that ZIKV affects neural stem cells function in both the human and mouse neocortex^8,28^; however, no systematic analysis has been done to demonstrate the effect of ZIKV on central nervous system development, including brain malformations and spinal cord dysfunction.

We conduct a longitudinal analysis by infecting pregnant mice with ZIKV at embryonic day 13.5 (E13.5) via *in utero* infection. The offspring were closely monitored from birth to P9 and into young adulthood P30. Here we showed that at the embryonic stage (E13.5–E17.5), ZIKV caused up to a 70% reduction in NSCs proliferation (E13.5–E15.5) compared to mock injection. Additionally, neurogenesis (E13.5–E17.5) was decreased significantly in the ZIKV group compared to that in the control group and was associated with a thinner cortex. Furthermore, cell death was found in the ZIKV-injected brain. At the P9 and young adulthood P30, we found that the body and brain weights were significantly decreased in the ZIKV group compared to those with mock infection. Also, at the young adulthood (P30), the hippocampus size was reduced, and the neurogenesis rate was decreased in the subventricular zone and dentate gyrus of the ZIKV-infected mouse hippocampus, the two neurogenic niche areas. The impaired neurogenesis was associated with a cognitive deficit in ZIKV-infected mice. However, the effect on brain and NSCs was immune to dengue virus at all stages, thus confirming that NSCs are more susceptible to ZIKV infection, which is unique to the brain.

Tremendous efforts have been made by different groups to recapitulate ZIKV infection in murine model either in adult^29,30^ or pregnant mice^11,31–33^. Irf3(−/−)Irf5(−/−)Irf7(−/−) triple knockout mice were generated to unresistant to ZIKV infection^29^. High viral loads were found in the brain and spinal cord in infected adult mice, causing neurodevelopment defects^29^. Miner *et al*.^32^ infected pregnant IFNαβR^−/−^ mice on E6.5–7.5 and found significant amount of virus in the pups’ brain, with reduction in body size. ZIKV infection in pregnant mice also cause abortion of some fetus. These results are consistent with our finding that ZIKV infection reduces body weight and affects spinal cord in pups. Recently, Julander *et al*.^34^ affect pregnant AG129 mice intraperitoneally and found lower survival rate, smaller size, shorter skull length in the offspring. Although ZIKV infection models have been established in these studies, only morphological consequences have been studied. Li *et al*.^11^ established a ZIKV infection model in mice via *in utero* injection into the lateral ventricle zone of the embryos. They found that ZIKV infection affect CNS development, including cell-cycle arrest, apoptosis, and thinner cortex during embryonic cortical development, in accordance with our findings. However, they failed to establish a post-natal mice model post-ZIKA infection to study the effect of ZIKV infection on CNS development after birth. In the current study, we have established a mice model carrying ZIKV from embryonic stage to adult stage. Comprehensive pathological effects including NPCs proliferation, differentiation, apoptosis, body size, brain weight, spinal cord, and muscle strength have studies before and after birth. Therefore, this study systematically examined pathological effects of ZIKV infection throughout the entire lifespan of mice, providing a comprehensive understanding of the pathologic effect of ZIKV on microcephaly.

Compared to other similar studies using *in utero* injection of ZIKV to mouse embryo brain, our dosage used is comparable or lower. Li *et al. in utero* injected 650 PFU of Zika virus into one side of the lateral ventricle of E13.5 mouse brains and detected high ZIKV RNA copies in embryo brain after 3–5 days post infection^11^. They found ZIKV infection disrupts NPCs development and leads to microcephaly. Similarly, Zhang *et al*. also used 650 PFU for *in utero* injection in mice and found upregulation of miR-9 is associated with microcephaly and ZIKV infection^35^. In addition, Julander *et al*. used a titter of 10^5.6^ TCID50 (in 0.1 ml, about 279,000 PFU) cell culture infectious dosage of Zika virus injected subcutaneously into AG129 mice to demonstrate that ZIKV can transmit through placental to fetus and has motor defects or cognitive deficits in offspring in AG129 mice^34^. Although it is not injected *in utero*, their dosage is much higher than the dosage we used (500 PFU). Therefore, we used a minimal ZIKV dosage to induce microcephaly pathology to avoid unnecessary dosage effect. Researchers also used ZIKV infection dosages, ranging from 10^3^ to 5 × 10^7^ PFU, on non-human primates subcutaneously or intramuscularly^36^.

However, to systematically study the effect of ZIKV infection on CNS from embryonic stage to whole lifespan in mice, we have to infect the embryonic mice during pregnancy. Zhang *et al*.^35^ and Li *et al*.^12^ *in utero* injected 650 PFU of Zika virus into one side of the lateral ventricle of E13.5 mouse brains to achieve embryo infection by ZIKV. To achieve ZIKV infection at embryonic stage and monitor the effects after birth, we determined ZIKV infection titre in comparison to DENV infection, ranging from 250 PFU to 20,000 PFU (Supplementary Fig. 1A). 500 PFU was choose to ensure both enough ZIKV infection dosage and minimal harm to embryos for their survival. The dosage we used (500 PFU) is also comparable with Zhang’s dosage (650 PFU) for *in utero* infection of ZIKV in mice.

To determine specific effects of ZIKV infection on mice, our study included mock (the ZIKV culture medium) and Dengue virus (same titre) as control for *in utero* injection. Body weight and brain weight of the pups were measured (Supplementary Fig. 1B–D). We found that ZIKV-infected pups have significantly lower (**p < 0.01) body weight than mock from P5 to P30, whereas no difference was detected in DENV infection compared to mock (Supplementary Fig. 1B). In addition, brain weight of ZIKV infection is significantly reduced (Fig. 1H–K), while brain weight of DENV infection is similar to mock control (Supplementary Fig. 1C,D). Given that DENV has no effect on brain development and neural stem cell fate (Supplementary Fig. 2G–K), our data indicates that the brain weight and body weight change, as well as the neurological defects are indeed due to ZIKV infection rather than a general effect. Furthermore, at E17.5, we found that both the immature neurons (Fig. 2E,F, marked by Tbr1) and mature neurons (Fig. 2G,H, marked by NeuN) are depleted upon ZIKV infection.

Interestingly, we found impaired spinal cord structure and function in the ZIKV group. For example, at P30, there are significantly fewer neurons in the anterior horn grey matter of the spinal cord in ZIKV group, as well as muscle dystrophy and significantly weaker forepaw grip strength. Two fatal cases of congenital ZIKV infection were reported to severely affect spinal cord in human^37^. ZIKV RNA was detected in their spinal cord samples and ZIKV proteins and viral particles were also found in cytoplasm of spinal neurons^37^. Spinal cord is distorted with markable neuron loss and infiltration of the grey matter by macrophage^37^. Fernandes *et al*. also found the spinal cord in neonatal mice infected with ZIKV subcutaneously after borne is injured with reduced astrocytes and prolonged cellular processes^38^. These data indicate that ZIKV infection either congenitally or experimentally can invade the peripheral nerve system and affects the morphology and normal function of spinal cord. Our Supplementary Fig. 1E further shows that ZIKA virus RNA copy is present more in spinal cord in young mice after *in utero* injection during embryonic stage than other organs such as kidney lung and liver. This suggests that Zika virus more preferentially transmits from CNS in the brain to the peripheral nerve system in the spinal cord. Our observations will prompt clinicians to pay more attention to spinal cord dysfunction during medical examination.

Moreover, footprint testing showed smaller strides and steps in ZIKV-infected mice, demonstrating a movement disorder. Our results demonstrate that *in utero* ZIKV infection during pregnancy not only leads to the development of microcephaly and brain damage, but also induces spinal cord neuron cell loss and functional disorder. These effects may also explain the muscle dystrophy and movement disorder phenotypes in babies infected with ZIKV during pregnancy^39^. Recently, Santos *et al*. reported a case, of which a patient with ZIKV infection exhibited post-encephalitic movement disorders, including parkinsonism and myorhythmia^40^. Smaller strides and steps found in ZIKV-infected mice is a good reflection of human movement disorder found in patients with microcephaly post ZIKV infection. Muscle weakness and movement disorder are classic symptoms in Guillain-Barré syndrome following infection with the Zika virus^39,41^. In addition, pathological damage in muscle is also found in AG129 mice post-ZIKV infection^42^. These effects may explain the muscle dystrophy and movement disorder phenotypes in babies infected with ZIKV during pregnancy^39,43^.

In the current study, we have systematically studied the effects of ZIKV infection on CNS and main organs in mice from embryonic stage to postnatal until death. We found ZIKV infection not only hinders CNS development leading to microcephaly but also affects spinal cord and body function such as muscle weakness after birth. The systematic study of ZIKV infection on mice throughout their whole lifespan will provide invaluable information on the effects of ZIKV on human throughout their entire life and also provide an accessible platform to model brain development for future testing treatments, including potential ZIKV antiviral drugs, miRNA therapy, and vaccines against CZS.

## Materials and Methods

### Mice

Time-mated C57BL/6 mice were purchased from InVivos (Singapore) at E13.5 for *in utero* injection of ZIKV and DENV. Mice were maintained in accordance with institutional guidelines, and all protocols were approved by the Institutional Animal Care and Use Committee (IACUC) of the Biological Resource Centre (BRC), Agency of Science, Technology & Research (A*STAR). The mice were maintained in a selective pathogen-free facility and exposed to a 12-hour light/dark cycle with food and water.

### Virus preparation

The virus strains used were the Zika virus (ZIKV strain PRVABC59, GenBank accession no. KU501215) and dengue virus serotype 2 (DENV2 strain 16681, GenBank accession no. NC_001474.2). Both ZIKV and DENV2 were propagated in baby hamster kidney (BHK) cells, and viral plaque assays were performed on monolayers of BHK cells in 24 well-plates. BHK cells were cultured in RPMI-1640 (HyClone) supplemented with 10% FBS (Capricorn Scientific), and the medium used for the propagation of viruses and the viral plaque assay was supplemented with 2% FBS. For the generation of ZIKV and DENV2 for animal challenge experiments, the infected culture supernatant was filtered through a 0.2-µm polyethersulfone (PES) membrane and stored at −80 °C.

Frozen tissues were weighed and transferred into homogenization tubes containing CK14 ceramic beads (Bertin Technologies), and 1 ml of RPMI-1640 supplemented with 2% FBS was added into each tube. Homogenization was performed at 6000 rpm for 10 seconds (s) using the Precellys®24 bead mill homogenizer (Bertin Technologies). Homogenates were clarified by centrifugation at 3500 × g for 10 minutes (min) at 4 °C, and the supernatants were further clarified by centrifugation at 10,000 × g for 10 min at 4 °C. The cleared supernatants were stored at −80 °C for downstream assays.

For the determination of the infectious virus titre by viral plaque assay, each tissue homogenate sample was 10-fold serially diluted, and 100 µl was added to monolayers of BHK cells in triplicate. The infection was allowed to proceed for 1 hour at 37 °C, 5% CO_2_ before the virus was removed. The cells were then washed to remove unbound virus particles with PBS (pH 7.4) and overlaid with medium containing 2% FBS and 1% carboxymethyl cellulose (CMC). The cells were incubated for 72 hours (h) for ZIKV and 120 h for DENV2, at 37 °C before they were fixed with 4% paraformaldehyde and stained with crystal violet. Plaques that formed were counted visually, and the infectious virus titre was calculated, expressed as the average number of PFU per microlitre (PFU μl^−1^) of samples.

For the detection of viral RNA in tissue samples, 140 µl of tissue homogenate was extracted using the QiaAmp Viral RNA Extraction Kit (Qiagen) according to manufacturer’s instructions. Reverse transcription was performed with 5 µl of extracted viral RNA with reverse primer (5′-TCCTGTGCCATTACGGTGAC-3′) and MMLV reverse transcriptase (Promega), and quantitative polymerase chain reaction (qPCR) was performed with the forward primer (5′-GACGTGGGAGTGCATACTATATGT-3′), the same reverse primer, and the SsoFast^TM^ EvaGreen® Supermix (Biorad) reagent, using the CFX96^TM^ Real-Time PCR Detection System. All reactions were conducted with a set of loading controls composed of 10-fold serially diluted viral RNA extracted from a virus sample titrated by viral plaque assay for quantitation of viral RNA copies.

### *In utero* injection

Timed-pregnant mice (E13.5) were anaesthetized with isoflurane (induction, 3.5%; surgery, 2.5%), and the uterine horns were exposed by laparotomy. The virus (approximately 2 µl, totally 500 PFU) was injected through the uterine wall into one of the lateral ventricles of each embryo with a glass capillary^21,22,44^. The uterine horns were placed back into the abdominal cavity after injection, and the embryos were allowed to continue their normal development. For the embryonic stage tests, embryo brains were harvested at E15.5 and E17.5 for the proliferation and differentiation analyses, respectively. For postnatal tests, the brain, spinal cord, and other organs of the pups were harvested at P9 and P30.

### RNA isolation and real-time PCR (RT-PCR)

Total RNA was extracted using mRNeasy kit (Qiagen) from tissue samples, followed by cDNA synthesis using a cDNA synthesis kit (Promega). PCRs were performed on three independent template sets, and cycling parameters were 94 °C for 15 s, 55 °C for 30 s, and 70 °C for 30 s for 40 cycles using the CFX96 real-time PCR detection system (Bio-Rad). For each assay, PCR was performed after a melting curve analysis. To reduce variability, we ran each sample in duplicate or even triplicate and included control qPCR reactions lacking the template for each run.

### EdU labelling and detection

An i.p. injection of 100 µg EdU (Invitrogen, C10340) in PBS was given to pregnant mice on E15.5; two hours later, samples were harvested for the proliferation experiment, and two days after that, samples were harvested for the labelled cell-tracing study. EdU was detected as described in the manufacturer’s protocol; in brief, cryostat brain sections were permeabilized in 0.5% Triton® X-100 in PBS at room temperature for 20 min. The sections were then washed twice with 3% BSA in PBS, followed by the addition of 0.5 ml Click-iT® reaction cocktail to the tissue and a 30-min incubation at room temperature with light protection. After the tissue was washed once with 3% BSA in PBS and then twice with PBS, the coverslips were mounted with Dako (S3023) fluorescent mounting medium and finally imaged and analysed with an Olympus (FV1000) confocal microscope.

### *In situ* cell death detection

The standard protocol was performed as described in the *In Situ* Cell Death Detection Kit (Roche, Cat. No. 11684795910). Simply, after the tissue sections were rinsed with PBS, the TUNEL reaction mixture was added to the sample, and the slides were incubated in a humidified atmosphere for 60 min at 37 °C in the dark, washed with PBS, and coverslip-mounted with Dako (S3023) fluorescent mounting medium. Finally, imaging and analysis was performed under an Olympus (FV1000) confocal microscope.

### Neuro Trace^TM^ fluorescent Nissl stain

The protocol was performed as described in the Nissl staining kit (N21479). Briefly, after the tissue sections were rehydrated with PBS, the tissue was permeabilized with 0.1% Triton® X-100 in PBS at room temperature for 10 min, washed, incubated with diluted Neuro Trace stain in PBS at room temperature for 20 min in the dark, washed again, and coverslip-mounted with Dako fluorescent mounting medium. Finally, imaging and analysis were performed with an Olympus (FV1000) confocal microscope.

### Nissl staining

The frozen tissue sections were dried at room temperature for 60 min and rinsed with PBS for two times. The slides were then rinsed in ddH2O for 1 min, dipped into Nissl stain (Cresyl violet solution) for 10 min, rinsed in ddH2O twice, dipped in 90%, 95% and 100% ethanol for 3 min each, then dipped in 100% ethanol again for 3 min, and dipped in two changes of 100% xylene for 3 min each. The slides were then mounted with DePex mounting medium (VWR 361254D), and images were taken with microscope.

### HE staining

The frozen tissue sections (6 μm) were dried at room temperature for 60 min and rinsed with PBS two times. The slides were then rinsed in ddH_2_O for 1 min, dipped into Hemaroxalinstain for 3 min, rinsed with ddH_2_O twice, dipped fast 8–12 times in acid ethanol, rinsed 2 × 1′ tap water, rinsed 1 × 2′ deionized water, 1 × 30 seconds Eosin, dipped in 90%, 95% and 100% ethanol for 3 min each, then dipped in 100% ethanol again for 3 min, and dipped in two changes of 100% xylene for 3 min each. The slides were then mounted with DePex mounting medium (VWR 361254D), and images were taken with an Olympus (IX83P2ZF) microscope.

### Immunohistochemistry, confocal imaging, and quantification

The mice were perfused and fixed with 4% paraformaldehyde in PBS overnight at 4 °C. The brains, spinal cord and other organs were removed and sectioned at 30 μm thickness with a Leica CM1950 cryostat (Leica). Slices were permeabilized with blocking solution containing 0.1% Triton X-100 and 1% BSA in PBS for 1 h at room temperature and then incubated with primary antibody overnight at 4 °C. The following day, after the slices were washed with PBS, they were incubated with fluorescently conjugated secondary antibodies (Invitrogen) and DAPI (Sigma) for 1 h at room temperature. The slices were then washed and mounted in Dako (s3023) mounting medium. Images of stained slices were acquired by a confocal microscope (Olympus FV1000) with various objective lenses. The images were analysed with ImageJ software. The following primary antibodies were used: mouse anti-flavivirus Envelope protein (AbFLAVENV-4G2), anti-Sox2 (Abcam/ab97959), anti-Tbr1 (Abcam/ab31940), anti-NeuN (Merk/ABN 78), anti-p-Histone H3 (Ser 10, Santa Cruz/sc-8656-R), anti-cleaved caspase-3 (Cell Signalling/Asp175), anti-Ki67 (Abcam/ab15580), and anti-doublecortin (Abcam/ab77450).

### Y-maze spontaneous alternation test

After the mice were introduced to the centre of the maze, the mice were allowed to freely explore the three arms for 5 min, amassing at least 15 arm entries. An entry occurred when all four limbs were within an arm. Spontaneous alternation (%) was defined as consecutive entries in 3 different arms, divided by the number of possible alternations (total arm entries minus 2)^45^. Re-entries into the same arm were rated as separate entries. Mice with less than 8 arm entries during a 5-min trial were excluded from the analysis.

### Footprint analysis

Mice will have their paws dipped in non-toxic water-based paints (fore paws in green, hind paws in red, CAS/MSDS needed). The mice were then placed at one end of an enclosed passageway (30 cm long) lined with white paper. Three trials will be performed on separate days within 1 week. Measurements will be made on two to four steps from the mid-portion of the run as follows: (1) Stride length was measured as the average distance of forward movement between each stride. (2) Distance from left or right front footprint/hind footprint overlap was used to measure uniformity of step alternation. At least nine steps would be measured for each mouse in total and the mean values will be computed.

### Statistical analyses

For each experiment, at least three independent experiments were performed. The images obtained from one representative experiment were presented. Statistical analysis was performed using SPSS. Data were represented as mean ± SD. For two-group comparisons, two-tailed Student’s t-test was performed. For multiple comparisons, data with a normal distribution were analysed by one-way ANOVAs followed by Tukey’s HSD test. The investigators were blinded to genotypes during experiments involving behavioural test and immunohistochemical counting. Significance level was set at *P* < 0.05.

## Supplementary information

Supplementary Figures

## Data Availability

All data generated or analysed during this study are included in this published article and its Supplementary Information Files.
