# Supplementary material for: In utero infection of Zika virus leads to abnormal central nervous system development in mice
Source: Sci Rep. 2019 May 13;9:7298. doi: 10.1038/s41598-019-43303-6 (PMC6513999; doi:10.1038/s41598-019-43303-6)
Supplement: Supplementary file 1 — Supplementary Figures [file 41598_2019_43303_MOESM1_ESM.pdf]

In utero infection of Zika virus leads to abnormal central nervous system development in mice

Wei Zhang[1], Yong Wah Tan[2], Wan Keat Yam[2], Haitao Tu[1], Lifeng Qiu[1], Eng King Tan[3,4,5], Justin Jang Hann Chu[2,6], Li Zeng[1,5,7*]

[1]Neural Stem Cell Research Lab, Research Department, National Neuroscience Institute, Singapore 308433.

[2]Collaborative Translation Unit for HFMD, Institute of Molecular and Cell Biology, Agency of Science, Technology & Research (A STAR), Singapore 138673.

[3]Research Department, National Neuroscience Institute, SGH Campus, Singapore 169856.

[4]Department of Neurology, National Neuroscience Institute, SGH Campus, Singapore 169856.

[5]Neuroscience & Behavioral Disorders Program, DUKE-NUS Graduate Medical School, Singapore 169857.

[6]Laboratory of Molecular RNA Virology and Antiviral Strategies, Department of Microbiology and Immunology, Yong Loo Lin School of Medicine, National University of Singapore, Singapore 117597.

[7]Lee Kong Chian School of Medicine, Novena Campus, 11 Mandalay Road, Singapore 308232

*** Corresponding Authors:**

Li Zeng, Li_Zeng@nni.com.sg

Running Title: *In utero* infection of ZIKV affects CNS development

Keywords: *In utero* injection; Zika virus (ZIKV); Central nervous system (CNS) development; microcephaly; mouse; neural stem cells (NSCs); spinal cord; Cortex

Supplementary Information

Supplementary Figure 1.

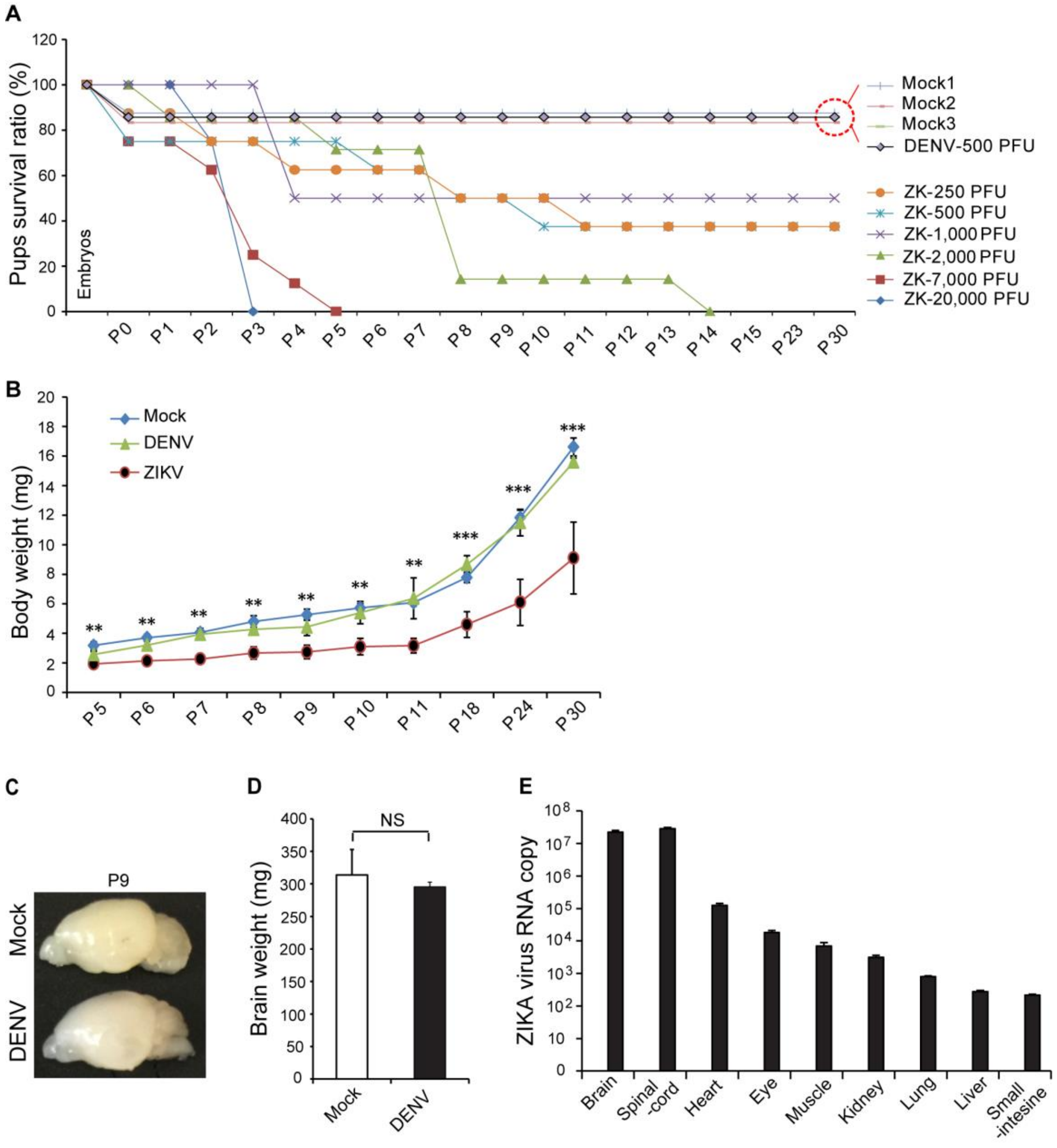

Supplementary Figure 1. ZIKV infection during pregnancy impairs postnatal brain development but DENV has no effect. **A**, Survival ratio of the pups after *in utero* injection of ZIKV at various doses at E13.5. **B**, Body weight changes of the pups in the mock, ZIKV (500 PFU) and DENV groups (500 PFU). **C,** Brain image and (**D**) brain weight at P9 after DENV infection (500 PFU, n = 3 per group). **E**, ZIKV detection by q-PCR in various organs at P9. ***P*< 0.01, ****P*< 0.001, calculated by one-way ANOVA with Tukey post hoc tests (**B**, **E**). NS, not significant. Graphs indicate mean ± SD.

Supplementary Figure 2.

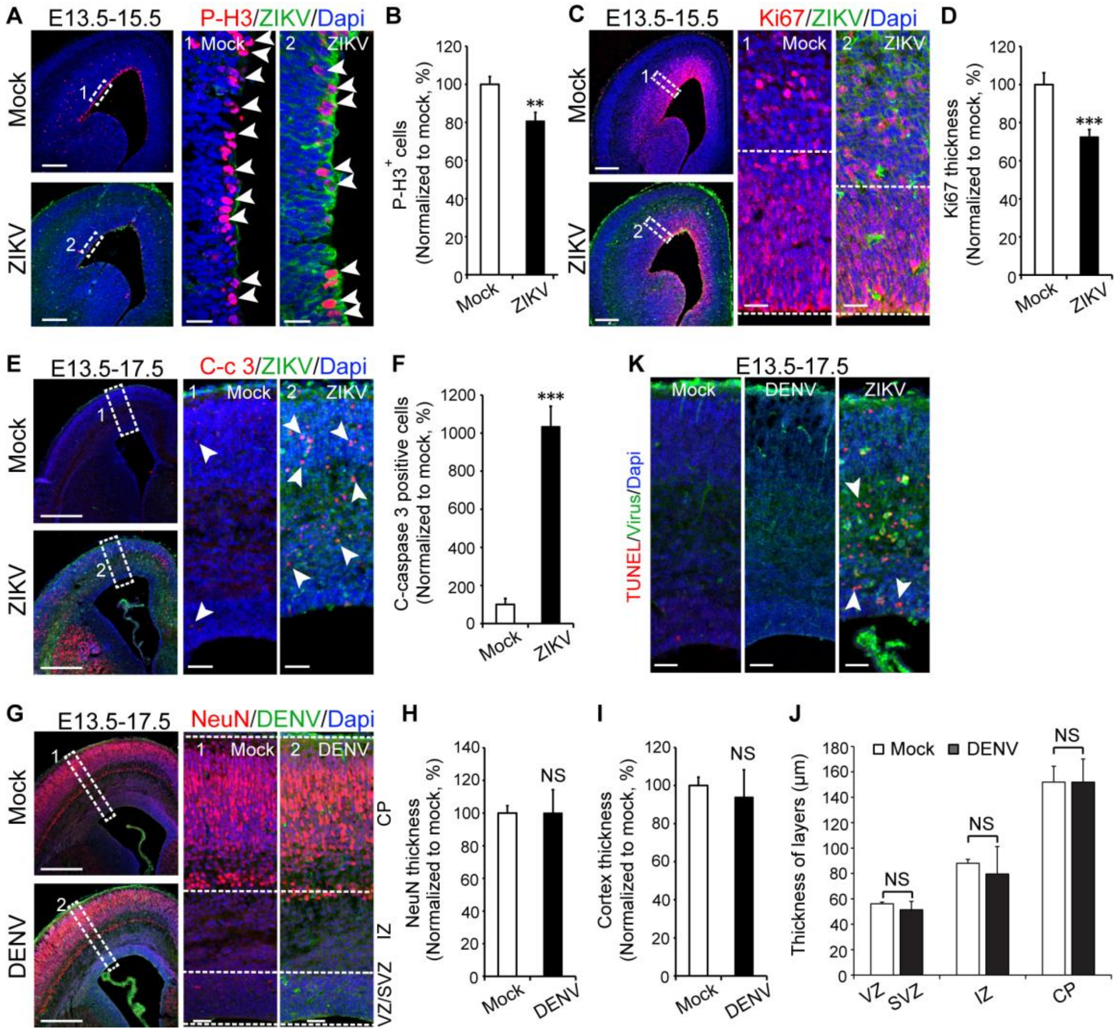

Supplementary Figure 2. ZIKV infection during pregnancy induces neurogenesis defect by affecting NSCs and cell death during the embryonic stage. **A**, Phospho-histone H3 staining and quantification of positive cells (**B**, n = 3 per group, >500 cells were counted) in brain sections at E15.5. Scale bar: left panel, 200 µm; enlarged images, 10 µm. **C,** Ki67staining and thickness calculation (**D**, n = 3 per group) at E15.5. Scale bar: left panel, 200 µm, enlarge images, 20 µm. **E**, Cleaved caspase-3 staining and quantification of positive cells (**F**, n = 5-6 per group, >500 cells were counted) at E17.5. Scale bar: left panel, 200 µm; enlarged images, 50 µm. **G**, NeuN staining of brain sections at E17.5 and analyses of NeuN thickness (**H**), cortex thickness (**I**), and thickness of different layers of the whole cortex (**J**, n = 5-6 per group). Scale bar: left panel, 200 µm; enlarged images, 50 µm. **K**, TUNEL staining of the brain cortex at E17.5; arrow head shows positive TUNEL staining signals. Scale bar:

50 µm. **P< 0.01, ***P< 0.001, calculated by Student's *t*-test. NS, not significant. Graphs indicate mean ± SD.

Supplementary Figure 3.

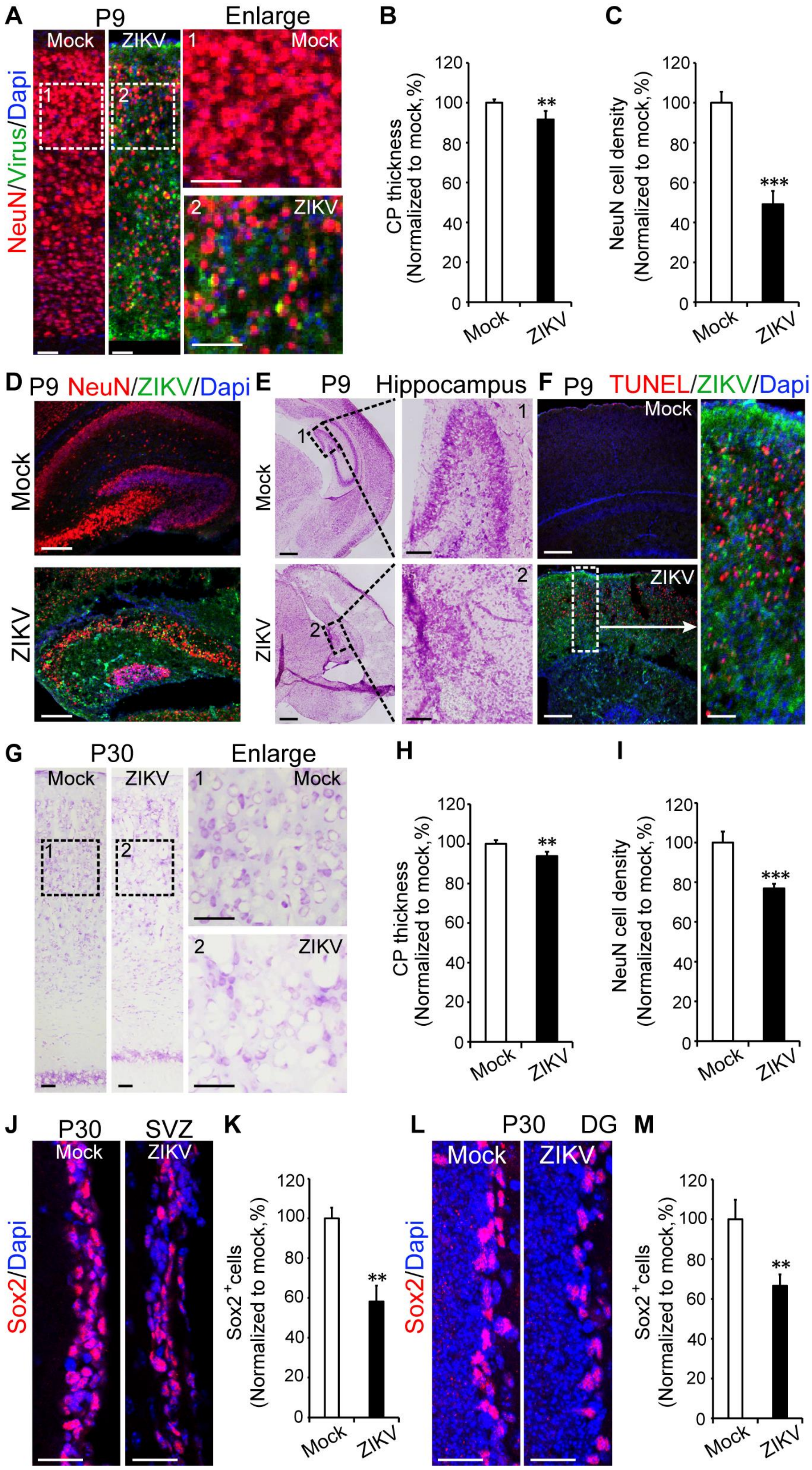

Supplementary Figure 3. ZIKV infection during pregnancy decreases neurogenesis in young adult mice. **A**, NeuN staining; (**B**) CP thickness and (**C**) neuron density in brain sections (n = 3-5 per group, >500 cells were counted) at P9; scale bar:50 µm. **D**, NeuN staining of the hippocampus at P9; scale bar: 50 µm. **E**, Nissl staining of the hippocampus at P9; scale bar: left, 500 µm; enlarged images, 50 µm. **F**, TUNEL and ZIKV staining of the brain cortex; scale bar: left, 100 µm; right, 50 µm. **G**, Nissl staining of brain cortex at P30 and (**H**, **I**) analyses of CP thickness and neuron density (**I**, n = 3-5 per group, >500 cells were counted); scale bar: 50 µm. **J**, Sox2 staining of the SVZ area and (**K**) quantification of Sox2-positive cells (n=3-5 per group, >500 cells were counted); scale bar: 500 µm. **L**, Sox2 staining of DG area and (**M**) quantification of Sox2-positive cells (n = 3-5 per group, >500 cells were counted) at P30. **$P< 0.01$, ***$P< 0.001$, calculated by Student's *t*-test. Graphs indicate mean ± SD.

Supplementary Figure 4.

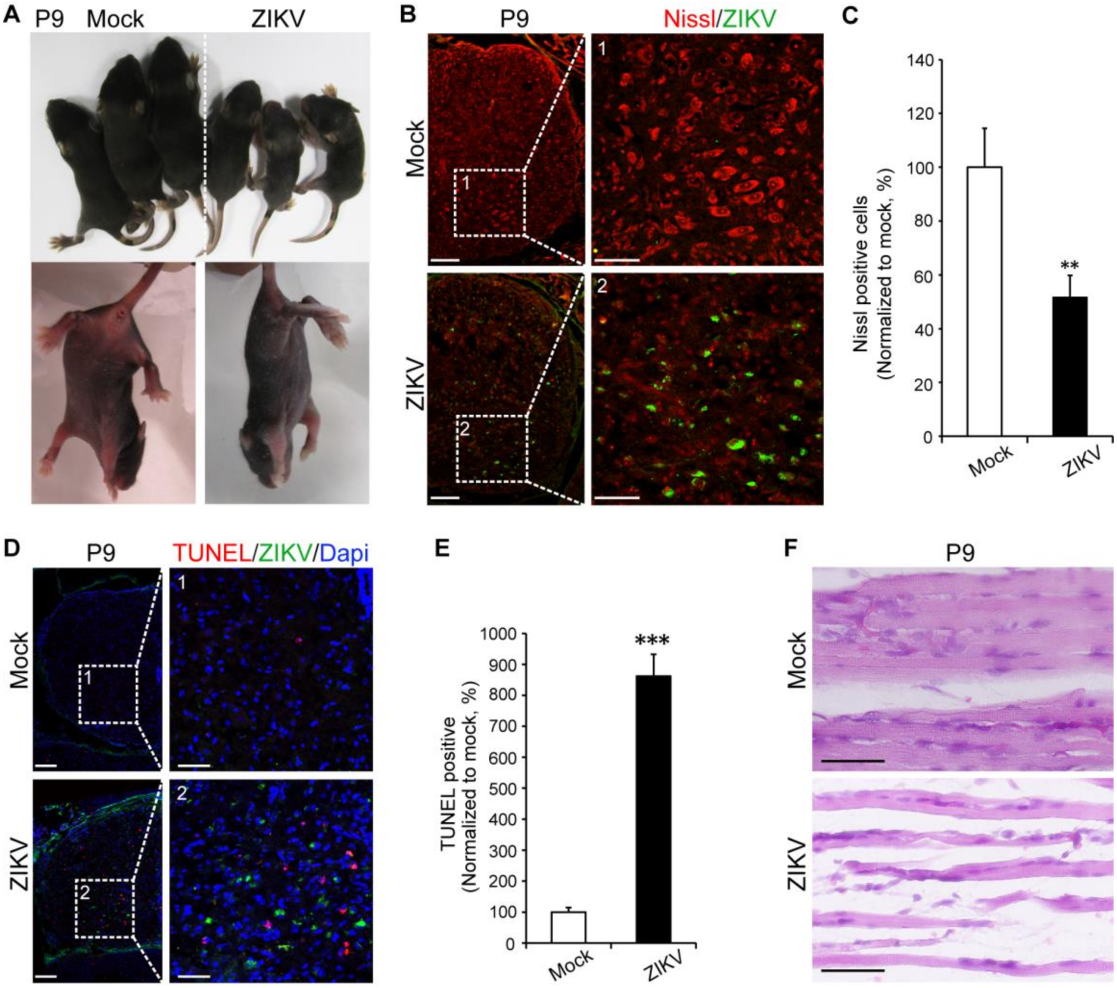

Supplementary Figure 4. ZIKV infection during pregnancy impairs postnatal spinal cord development and function. **A**. ZIKV and mock infected mice at P9. **B**. Fluorescent Nissl staining and quantification of positive neuronal cells in the anterior horn of grey matter (**C**, n = 3-5 per group, >500 cells were counted) at P9. **D**, TUNEL staining of the spinal cord and (**E**) quantification of the positive signal (n = 3-5 per group, >500 cells were counted) at P9. **F**, HE staining of the hind limb muscle at P9. **P< 0.01, ***P< 0.001, calculated by Student's t-test. Graphs indicate mean ± SD, Scale bar: B and D, left panel, 200 μm; right enlarged images, 50 μm. F, 50 μm.

Supplementary Figure 5.

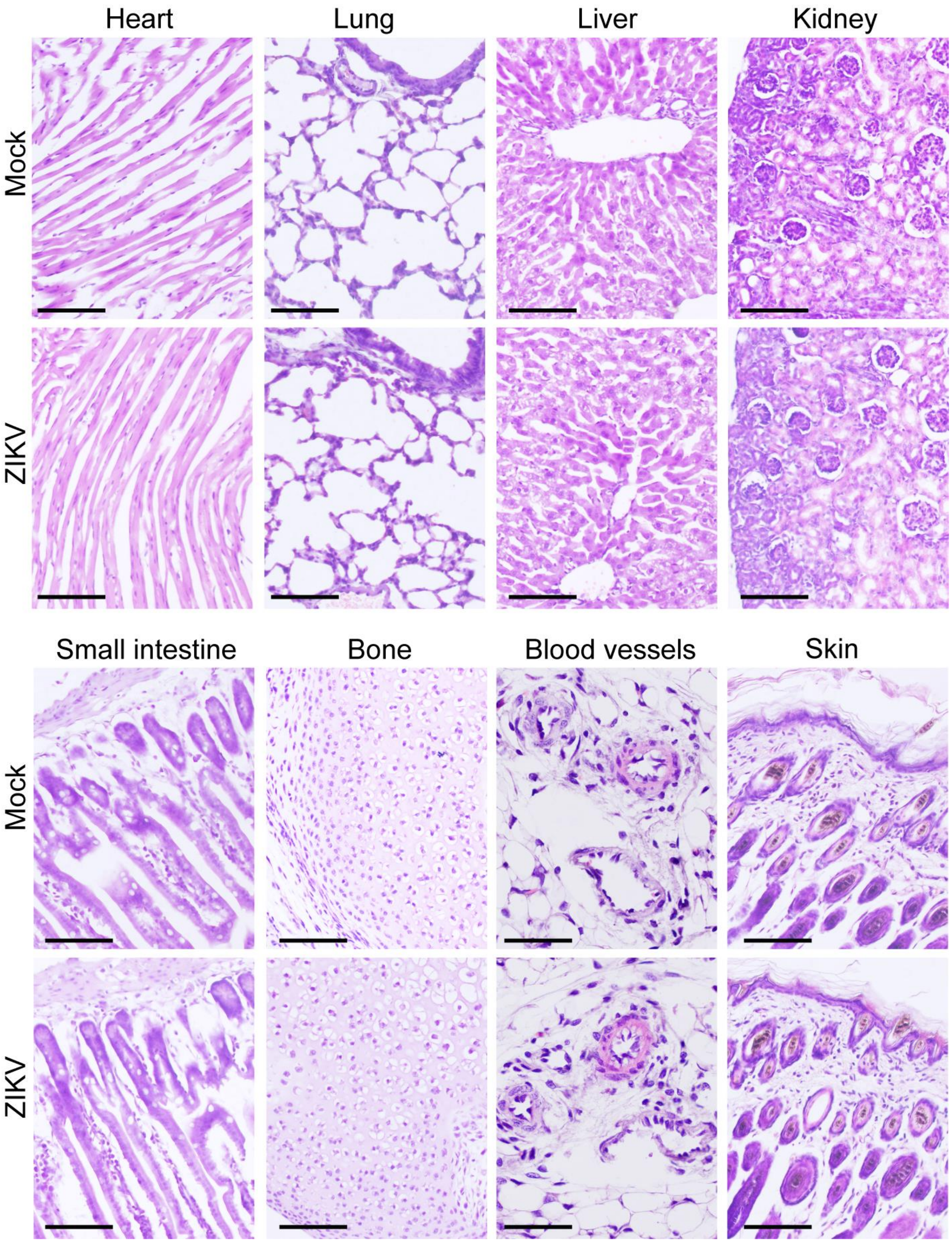

Supplementary Figure 5. HE staining of different organs at P30. HE staining of different organs in heart, lung, liver, kidney, small intestine, bone, blood vessels, and skin at P30. Scale bar, 50 μm.
